# Risk factors associated with house entry of malaria vectors in an area of Burkina Faso with high, persistent malaria transmission and high insecticide resistance

**DOI:** 10.1186/s12936-021-03926-5

**Published:** 2021-10-10

**Authors:** Jean Baptiste Yaro, Alfred B. Tiono, Antoine Sanou, Hyacinthe K. Toe, John Bradley, Alphonse Ouedraogo, Z. Amidou Ouedraogo, Moussa W. Guelbeogo, Efundem Agboraw, Eve Worrall, N.’Fale Sagnon, Steven W. Lindsay, Anne L. Wilson

**Affiliations:** 1 Centre National de Recherche Et de Formation Sur Le Paludisme, Ouagadougou, Burkina Faso; 2 Department of Biosciences, Durham University, Durham, UK; 3 MRC International Statistics and Epidemiology Group, London School of Hygiene and Tropical Medicine, London, UK; 4 Department of Vector Biology, Liverpool School of Tropical Medicine, Liverpool, UK

## Abstract

**Background:**

In rural Burkina Faso, the primary malaria vector *Anopheles gambiae *sensu lato (*s.l*.) primarily feeds indoors at night. Identification of factors which influence mosquito house entry could lead to development of novel malaria vector control interventions. A study was therefore carried out to identify risk factors associated with house entry of *An. gambiae s.l*. in south-west Burkina Faso, an area of high insecticide resistance.

**Methods:**

Mosquitoes were sampled monthly during the malaria transmission season using CDC light traps in 252 houses from 10 villages, each house sleeping at least one child aged five to 15 years old. Potential risk factors for house entry of *An. gambiae s.l.* were measured, including socio-economic status, caregiver’s education and occupation, number of people sleeping in the same part of the house as the child, use of anti-mosquito measures, house construction and fittings, proximity of anopheline aquatic habitats and presence of animals near the house. Mosquito counts were compared using a generalized linear mixed-effect model with negative binomial and log link function, adjusting for repeated collections.

**Results:**

20,929 mosquitoes were caught, of which 16,270 (77.7%) were *An. gambiae s.l.* Of the 6691 *An. gambiae s.l*. identified to species, 4101 (61.3%) were *An. gambiae *sensu stricto and 2590 (38.7%) *Anopheles coluzzii*. Having a metal-roof on the child’s sleeping space (IRR = 0.55, 95% CI 0.32–0.95, p = 0.03) was associated with fewer malaria vectors inside the home.

**Conclusion:**

This study demonstrated that the rate of *An. gambiae s.l.* was 45% lower in sleeping spaces with a metal roof, compared to those with thatch roofs. Improvements in house construction, including installation of metal roofs, should be considered in endemic areas of Africa to reduce the burden of malaria.

**Supplementary Information:**

The online version contains supplementary material available at 10.1186/s12936-021-03926-5.

## Background

**Fig. 1:**
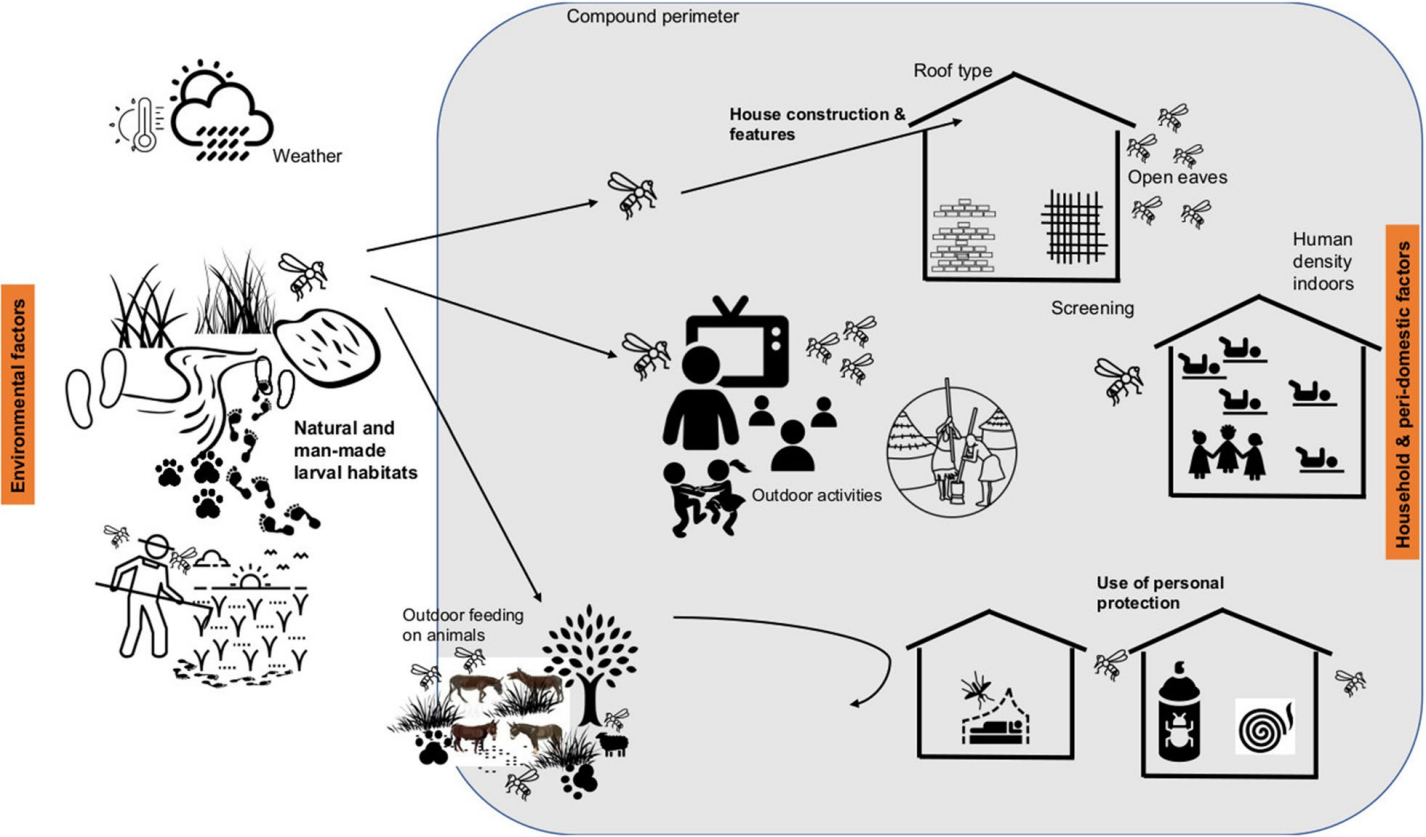
Environmental and household factors affecting the abundance of malaria vectors indoors. Indoor malaria vector abundance is affected by environmental risk factors such as weather conditions, proximity and productivity of natural and human-made larval habitats, presence of livestock and animals that may divert or attract malaria vectors, outdoor activities such as cooking, sleeping or playing which may increase biting (especially where outdoor early evening biting is a problem). Indoor malaria vector density can be reduced by features of the house construction (e.g. screening, closed eaves) and by use of personal protective measures such as ITNs and household insecticides. Increased human density indoors increases the odour plume of carbon dioxide and other attractants which can attract malaria vectors towards an inhabited house

Despite large reductions in the malaria burden across sub-Saharan Africa from 2000 to 2015 [1], some countries continue to experience extremely high malaria transmission [2]. In Africa, malaria transmission is highly efficient because of the wide distribution of *Anopheles gambiae *sensu lato (*s.l*.), an effective malaria vector that readily feeds on people indoors at night, where about 79% of malaria transmission typically occurs [3]. The indoor density of malaria mosquitoes is dependent on numerous environmental and household factors, including the abundance and proximity of aquatic habitats of malaria mosquitoes [4, 5], presence of large domesticated animals who may serve as alternative blood sources [6], typology of houses [7, 8], use of anti-mosquito measures in the house [5], number of residents [9] and variability in the attractiveness of individual people [10] (Fig. 1).

Burkina Faso is an area of intense seasonal malaria transmission, and cases are increasing [11–13] despite high coverage of vector control tools, including three national insecticide-treated net (ITN) mass distribution campaigns in 2010, 2013 and 2016 [14]. Resistance to pyrethroids, the main insecticide class used for treating ITNs, is high in *An. gambiae s.l*., and research conducted in the study area suggests that exposure to ITNs may have no impact on the lifelong survival of malaria vectors [15]. New tools are urgently needed to reduce the burden of malaria in Burkina Faso and other countries in sub-Saharan Africa.

Several studies have demonstrated that malaria mosquito house entry can be reduced through simple changes to house design, such as closing eaves and screening windows and doors [16]. The use of personal protective measures such as ITNs and spatial repellents may also reduce transmission [17, 18]. There is a lack of evidence, of whether such methods will reduce house entry of malaria vectors in settings of high insecticide resistance, such as the study site in south-west Burkina Faso. A risk factor survey was conducted to identify variables associated with indoor density of *An. gambiae s.l*. during the malaria transmission season in an area of intense malaria transmission in south-west Burkina Faso. Findings from this study might identify potential opportunities for improving malaria control in Burkina Faso and other countries in sub-Saharan Africa experiencing persistently high malaria transmission.

## Methods

### Study site

**Fig. 2:**
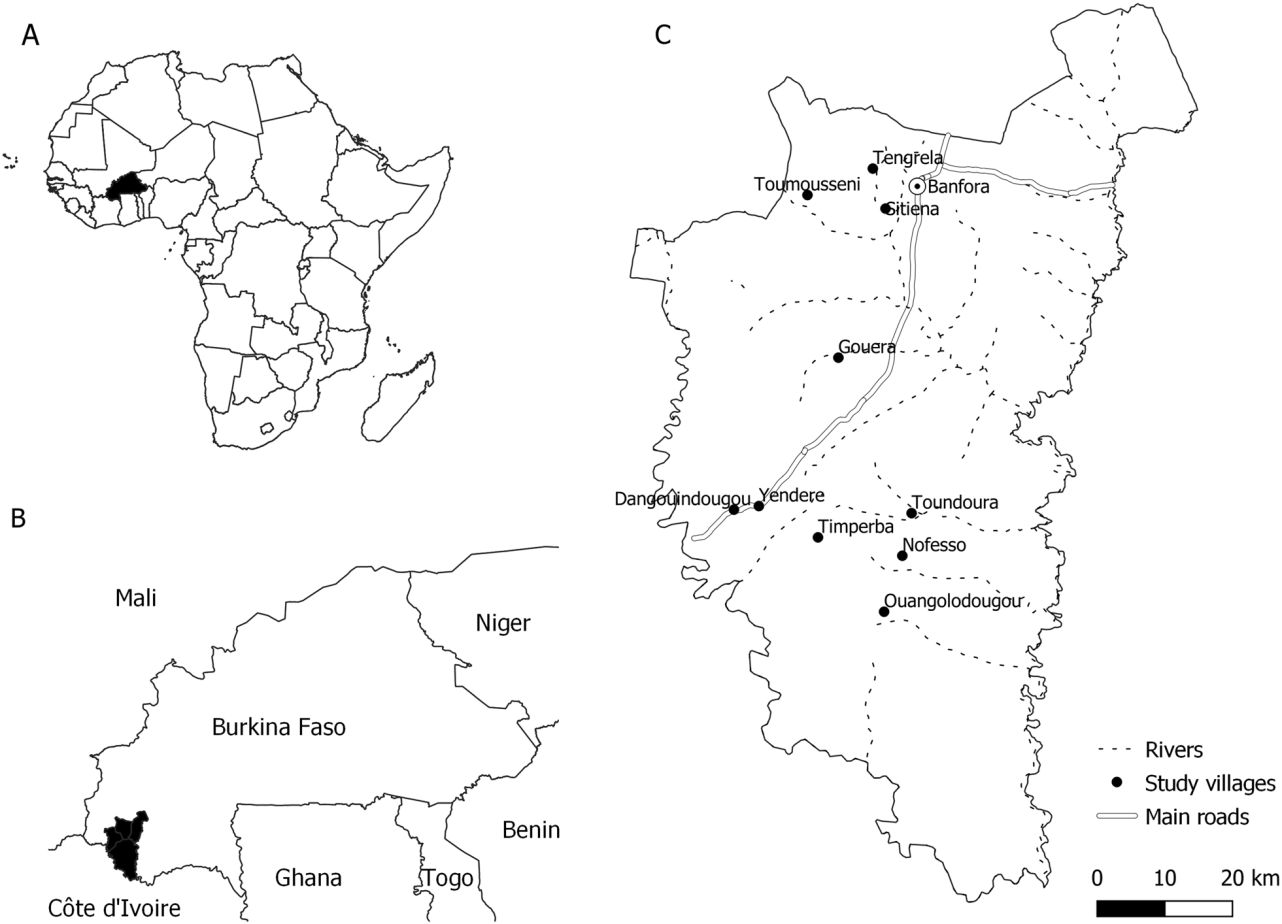
Map of study site. **A** location of Burkina Faso; **B** location of study site in Burkina Faso; **C** location of study villages in study site

The study was conducted in Banfora Health District, in the Cascades Region, south-west Burkina Faso (Fig. 2). This is an area of Sudanian savannah covering 6295 km^2^ with an estimated population of 407,073 inhabitants [13]. Malaria transmission is intense and seasonal, occurring mainly during the rainy season, from May to November [19]. *Plasmodium falciparum* accounts for 90% of cases [19]. The main malaria vectors are *An. gambiae *sensu stricto (*s.s*.) and *Anopheles coluzzii* [20]. In 2016, approximately 1 year before this study took place, a universal coverage campaign distributed ITNs with permethrin or deltamethrin (Sumitomo Chemical, Vestergaard and BASF) at a rate of one net for every two people at risk. No additional ITNs were distributed by the study. No indoor residual spraying was conducted. Families typically live alongside their extended family in compounds, each led by a compound head.

Compounds typically consist of multiple single room buildings arranged in a circular or semi-circular fashion around a shared open space, with sleeping areas, kitchens and toilets existing as separate structures [21]. Polygyny is common, with multiple wives and their children often living in the same compound. Children typically sleep with their mother but once they are old enough (~ 10 years) boys and girls are separated and move into another single room house in the compound.

### Study design

The study was nested in a cohort study of risk factors for *P. falciparum* infection in children aged five to 15 years [22]. This study reports on the household and environmental risk factors associated with the density of *An. gambiae s.l.* in the children’s sleeping space during the peak malaria transmission period from 24 July to 28 December 2017.

### Recruitment of study cohort

Sampling and recruitment of the study cohort is described elsewhere [23]. In brief, a random sample of 10 villages were selected from a list of villages in the study area using a two-stage process. Firstly, five health centres in the study area were selected, each with a catchment radius of 10 km. Secondly, two villages, at least 3 km apart, were selected from each catchment area. An enumerated list of children in the study villages was obtained from the Banfora Demographic and Health Surveillance System. From each village, a random sample of 30 children aged 5 to 15 years were chosen. Each child was selected from a separate house, and, where possible, a separate compound. Children were included in the study if they were of the appropriate age, were likely to remain resident in the village over the duration of the transmission season and the caregiver provided written informed consent to participate in the study. Children received a curative dose of artemisinin-based combination therapy and 252 children who were successfully cleared of *P. falciparum* infection (confirmed by polymerase chain reaction, PCR) were included in the cohort study and this current study reports on the entomological surveillance from the children’s sleeping space.

### Entomological surveillance

CDC light traps (John Hock, Gainsville, USA) were used to estimate indoor mosquito densities in the study child’s sleeping space. These traps were placed with the bulb 1.5 m above the floor, approximately 0.5 m from the foot end of a bed with an ITN occupied by the study child. Houses were sampled from 19.00 h to 06.00 h every 4 weeks. Two villages (Nofesso and Ouangolodougou) were inaccessible for two weeks at the start of the study period due to flooding. Mosquitoes were taken to the laboratory in cool boxes and killed by freezing. Mosquitoes were identified morphologically using established keys [23]. The presence of circumsporozoites protein (CSP) in *An. gambiae s.l.* were identified using an enzyme-linked immunosorbent assay [24] and *An. gambiae s.l*. females were typed to species by PCR [25, 26]. If less than 100 *An. gambiae s.l.* were caught per house then all were typed to species by PCR, but if the number was greater than 100, a third of the mosquitoes were randomly sampled for PCR analysis.

### Risk factor assessment

In June, a questionnaire was administered to the caregiver of the study child to collect information on ethnicity, education level and occupation of caregivers, ITN use during the previous night, use of other protective measures (e.g. insecticide knockdown spray, mosquito coils, traditional spatial repellent), number of people sleeping in the same part of the house as the study child, roof, wall and floor construction of the child’s sleeping space, whether the eaves (the gap between the top of the wall and the roof) were open or closed and presence of mosquito screening. Information was also collected from the head of the child’s household (typically the child’s father) on asset ownership and household characteristics, following standard procedures used in the Burkina Faso Demographic and Health Survey (DHS questionnaire, Additional File 1) [27]. The DHS questionnaire specifically referred to the construction of the head of household’s house, which may or may not have reflected the construction of the study child’s sleeping space due to the social structure in the study area. The number and type of large domestic animals (cattle, goats, sheep, pig, dog, donkeys or horses) tethered within 5 m of the sleeping space was recorded. The sleeping space was geo-located using a handheld global positioning system (GARMIN eTrex 20). Larval surveys were carried out in each village in September, during the peak of the transmission season. All water bodies within 1 km of the sleeping space were mapped, including irrigated fields, streams and ponds, puddles, and foot or hoof prints. The presence of anopheline larvae was recorded with a dipper.

### Data management and statistical analysis

Data were collected on Android personal digital assistants programmed using the KoboCollect system and included drop down boxes and consistency checks to reduce data entry errors. Following cleaning, the dataset was locked and saved in Microsoft Access. The primary outcome was the number of female *An. gambiae s.l*. collected in each child’s sleeping space per night. QGIS Geographic Information System (QGIS Development Team (2019), Open Source Geospatial Foundation Project) was used to determine distances between the child’s sleeping space and aquatic habitats. Principal component analysis (PCA) was used to calculate the socio-economic status (SES) factor score of the head of the child’s household. SES factor scores were ranked, and households divided into five equal wealth quintiles, from 1, the poorest, to 5, the least poor. The entomological inoculation rate (EIR) or estimated number of infectious bites per study child during the transmission season was calculated using the formula EIR = *MaSd* where *Ma* is the human biting rate, estimated from the arithmetic mean number of female *An. gambiae s.l.* caught per light trap night across the transmission season, where *S* is the proportion of female *An. gambiae s.l.* found to be CSP positive by village and *d* is the number of days in the transmission season. Mean values were compared using a t-test and proportions compared using chi-squared tests. A generalized linear mixed-effect model with a negative binomial distribution, to account for overdispersion, and log link function was used to identify risk factors associated with the mean number of *An. gambiae s.l.* per catch night per sleeping space each month. Risk factors were selected a priori based on importance for malaria vector house entry. These were SES quintile of the household head, ITN use, use of other protective measures, number of people sleeping in the same part of the house as the child, roof, floor and wall material in the sleeping space, eaves (open or closed), presence of large domesticated animals within 5 m of the sleeping space and presence of habitats positive for anopheline larvae within 300 m of the child’s sleeping space. A random effect for study child ID number was used to account for repeated measures on the same sleeping space and village was included as a fixed effect. Univariate analysis was conducted followed by construction of a simple multivariate model in which every risk factor was included, irrespective of whether the variable was significant in the univariate model. Interactions were tested between a subset of variables that were thought to be biologically relevant to explore. Means and 95% confidence intervals were calculated. Statistical analysis was carried out in Stata 15 (Statacorp, Texas, USA). The study is reported following STROBE guidelines [28].

## Results

As reported elsewhere [22], a total of 20,929 mosquitoes were caught from 1151 trap collections in 252 children’s sleeping spaces, with 16,270 of these being *An. gambiae s.l.* (77.7%). Of the 6691 *An. gambiae* s*.l.* identified to species (excluding 924 lost and non-identified samples), 4101 were *An. gambiae s.s.* (61.3%) and 2590 *An. coluzzii* (38.7%). Malaria vector abundance rose in July after the start of the rains in May, reaching a peak in August, before declining to low levels in November and December. 3.3% of *An. gambiae s.l.* were CSP positive and the overall EIR in the study area was 80.4 infective bites/child over the six-month transmission season. The village-level EIR ranged from 40.8 in Timperba to 191.9 in Tondoura.

The ethnic composition of the study population was Gouin (38.9%, 98/252), Karaboro (21.8%, 55/252), Mossi (11.5%, 29/252), Turka (9.1%, 23/252), Fulani (6.3%, 16/252), Senoufo (4.4%, 11/252) and other ethnic groups (7.9%, 20/252; Table 1). Caregivers were predominantly illiterate (79.0%, 199/252) and farmers (95.2%, 240/252). 80.6% (203/252) of caregivers reported that their child slept under an ITN the previous night, while 15.9% (40/252) reported using mosquito coils and 6.4% (16/252) insecticide knockdown spray. Children’s sleeping spaces were constructed with predominantly brick walls (57.9%, 146/252), cement or tiled floors (70.6%, 178/252), metal roofs (75.8%, 191/252) and open eaves (54.8%, 138/252). Window screening was rare (0.4%, 1/252). 67.1% (169/252) of households had large domestic animals (cattle, goats, sheep, dogs, pig, donkeys or horses) within 5 m of the house. 50.4% (127/252) of child’s sleeping spaces were located within 300 m of an aquatic habitat containing anopheline larvae.

**Table 1:** Characteristics of the study children and their sleeping spaces

Characteristic		Number (%) N = 252
Socio-demographic characteristics		
Ethnicity	Gouin	98 (38.9%)
	Karaboro	55 (21.8%)
	Mossi	29 (11.5%)
	Turka	23 (9.1%)
	Fulani	16 (6.3%)
	Senoufo	11 (4.4%)
	Others	20 (7.9%)
Caregivers education level	Illiterate	199 (79.0%)
	Primary school	45 (17.9%)
	Secondary school or above	8 (3.2%)
Caregivers occupation	Farmer	240 (95.2%)
	Non-farmer	12 (4.8%)
Number of people sleeping in the same part of the house as the study child (including child)	≤ 6	55 (21.8%)
	7–12	118 (46.8%)
	> 12	79 (31.3%)
Use of personal protective measures		
Reported ITN use	Used ITN usually	215 (85.3%)
	Used an ITN the previous night	203 (80.6%)
Use of other personal protection methods	Coils	40 (15.9%)
	Insecticide spray	16 (6.4%)
	Traditional spatial repellent	2 (0.8%)
	None	184 (73.0%)
Construction of child’s sleeping space		
Roof material	Non-metal (Thatch/mud)	52 (20.6%)
	Metal	191 (75.8%)
Wall material	Mud	65 (25.8%)
	Brick	146 (57.9%)
	Cement blocks (plastered or painted)	32 (12.7%)
Floor material	Mud	65 (25.8%)
	Cement/tile	178 (70.6%)
Eave status	Open	138 (54.8%)
	Closed	102 (40.5%)
Window screening	Absent	242 (96.0%)
	Present	1 (0.4%)
Environmental factors		
Presence of large domestic animals within 5 m of the sleeping space	Present	169 (67.1%)
	Absent	80 (31.7%)
Proximity of sleeping space to anopheline positive larval habitats	< 300 m	127 (50.4%)
	≥ 300 m	125 (49.6%)

Sleeping spaces with metal roofs were more likely to have walls and floors made of finished materials and open eaves than thatch roof sleeping spaces. 81.7% (156/191) of sleeping spaces with a metal roof had a cement or tiled floor compared to 42.3% (22/52) of those with a thatch roof (p < 0.001). Metal roof sleeping spaces were also more likely to have brick or cement walls (78.0%, 149/191) compared to thatch roof sleeping spaces (55.8%, 29/52, p < 0.001). Sleeping spaces with a metal roof were also more likely to have open eaves (66.0%, 126/191) than sleeping spaces with a thatch roof (23.1%, 12/52 and 28.8%, 15/52 respectively, p < 0.001 and p = 0.003). Children living in sleeping spaces with a thatch roof were more likely to share the same part of the house with greater than 12 people (38.5%, 20/52), than children living in sleeping spaces with a metal roof (26.2%, 50/191; p < 0.001). There was no association between metal roof sleeping spaces and distance from the nearest anopheline larvae positive habitat (99/191, 51.8% of those in metal roof houses lived within 300 m of a positive anopheline habitat, *versus* 23/52, 44.2% of those in thatch roof houses, p = 0.38).

There did not appear to be any strong trend between SES quintile of the household head and roof material, floor material or wall material of the child’s sleeping space (Table 2). Children living in poorer households were, however, more likely to have open eaves in their sleeping space (78.3% of quintile 1) than richer households (26.7% of quintile 5; p < 0.001).

**Table 2:** Construction details of study child’s sleeping space by socio-economic status of the household head

Construction feature of the child’s sleeping space	Socio-economic status of household head*					P value
	Poorest (N = 46) n (%)	Poor (N = 44) n (%)	Middle (N = 44) n (%)	Rich (N = 46) n (%)	Richest (N = 47) n (%)	
Roof material						
Non-metal (thatch)	7 (15.2)	9 (20.9)	5 (11.9)	14 (31.8)	11 (24.4)	0.17
Metal	39 (84.8)	34 (79.1)	37 (88.1)	30 (68.2)	34 (75.6)	
Floor material						
Mud	5 (10.9)	17 (39.5)	8 (19.0)	14 (31.8)	13 (28.9)	0.02
Cement/tile	41 (89.1)	26 (60.5)	34 (81.0)	30 (68.2)	32 (71.1)	
Wall material						
Mud	13 (28.3)	12 (27.9)	7 (16.7)	16 (36.4)	12 (26.7)	0.14
Brick	29 (63.0)	26 (60.5)	29 (69.0)	18 (40.9)	30 (66.7)	
Cement blocks	4 (8.7)	5 (11.6)	6 (14.3)	10 (22.7)	3 (6.7)	
Eaves						
Open	36 (78.3)	32 (76.2)	25 (62.5)	19 (43.2)	12 (26.7)	< 0.001
Closed	10 (21.7)	10 (23.8)	15 (37.5)	25 (56.8)	33 (73.3)	

*SES missing for 25 study children

In the final multivariate model, having a metal roof (IRR = 0.55, 95% CI 0.32–0.95, p = 0.03) was associated with fewer malaria vectors indoors, after adjusting for the other risk factors including SES of the household head (Table 3). There was no association between malaria vector density and SES of the household head, use of ITNs or other personal protection measures, the number of people living in the same part of the house as the study child, floor or wall material, eave status, presence of domestic animals within 5 m or presence of anopheline positive larval habitats within 300 m of the child’s sleeping space.

**Table 3:** Risk factors for *An. gambiae s.l.* abundance in study children’s sleeping space

Variable	Mean mosquito density per month (95% CI)	Univariate analysis		Multivariate analysis	
		IRR (95% CI)	P value	IRR (95% CI)	P value
Socio-economic status of household head					
Poorest	23.0 (10.0–36.1)	1		1	0.67
Poor	14.1 (6.8–21.3)	0.78 (0.42–1.45)	0.37	0.73 (0.39–1.37)	
Middle	11.8 (7.7–15.9)	0.91 (0.49–1.69)		0.89 (0.48–1.66)	
Rich	11.5 (7.3–15.8)	0.74 (0.38–1.43)		0.82 (0.42–1.63)	
Richest	12.4 (5.6–19.1)	0.67 (0.30–1.51)		0.73 (0.32–1.65)	
ITN use the previous night					
No	7.1 (4.5–9.8)	1		1	
Yes	15.8 (11.7–19.8)	1.18 (0.62–2.26)	0.62	1.12 (0.56–2.26)	0.75
Use of other personal protection measures (insecticide knockdown spray, mosquito coils, traditional spatial repellent)					
No	15.8 (11.4–20.2)	1		1	
Yes	9.6 (6.3–13.0)	0.95 (0.56–1.59)	0.83	1.02 (0.58–1.79)	0.95
Number of people sleeping in the same part of the house as the study child					
≤ 6	13.5 (9.6–17.4)	1		1	
7–12	17.1 (10.3–23.8)	1.10 (0.65–1.85)	0.73	1.33 (0.75–2.36)	0.52
> 12	10.4 (7.5–13.3)	0.77 (0.45–1.31)	0.33	0.83 (0.45–1.53)	
Roof material of child’s sleeping space					
Non-metal (thatch)	14.3 (8.1–20.6)	1		1	
Metal	14.4 (10.3–18.4)	0.58 (0.36–0.94)	0.03	0.55 (0.32–0.95)	0.03
Floor material of child’s sleeping space					
Mud	17.4 (11.4–23.3)	1		1	
Cement/tile	13.3 (9.1–17.5)	0.64 (0.41–0.99)	0.04	0.70 (0.42–1.20)	0.20
Wall material of child’s sleeping space					
Mud	17.4 (11.3–23.4)	1		1	
Brick	13.6 (8.5–18.6)	1.01 (0.63–1.62)	0.97	1.18 (0.67–2.07)	0.57
Cement	11.6 (7.0–16.1)	0.82 (0.40–1.69)	0.59	0.98 (0.44–2.18)	0.97
Eaves of child’s sleeping space					
Open	14.9 (9.6–20.3)	1		1	
Closed	13.8 (9.8–17.7)	1.00 (0.62–1.60)	0.99	0.95 (0.57–1.58)	0.84
Presence of large domestic animals within 5 m of the sleeping space					
Present	14.9 (10.1–19.7)	1		1	
Absent	12.7 (9.3–16.0)	1.12 (0.76–1.67)	0.56	1.12 (0.74–1.69)	0.60
Distance of sleeping space to positive larval habitat					
< 300 m	9.5 (7.3–11.8)	1		1	
> 300 m	18.6 (12.4–24.9)	1.54 (1.04–2.30)	0.03	1.43 (0.93–2.19)	0.11

## Discussion

The study findings demonstrate highly intense transmission of malaria in Banfora Health District with a person sleeping without an ITN experiencing a seasonal EIR varying from 40.8 infectious bites per person in Timperba village to 191.9 in Toundoura village [27]. Reported ITN use was high with 80.6% of caregivers reporting that the study child slept under an ITN the previous night. The incidence rate of *An. gambiae s.l.* in metal roof sleeping spaces was almost half that in thatch roof houses (IRR = 0.55, 95% CI 0.32–0.95, p = 0.03) and no other significant risk factors were identified.

Finding fewer malaria vectors indoors in metal roof houses compared to thatch roof houses may be a result of the indoor climate of the different typologies of houses. Metal roof houses tend to be hotter and less humid than thatch roof houses which can reduce the survivorship of malaria vectors resting indoors [29]. Alternatively, metal-roofs may simply be a marker for a better-quality home that is less porous to mosquitoes since metal roof houses are often better built, with fewer mosquito entry points, than thatched-roofed houses. This study found metal-roof houses were more likely to have floors and walls made of finished materials, than thatch-roof houses, although the proportion of metal roof houses with open eaves was higher. This was an unusual finding since metal roofs and closing of the eaves are often implemented together.

Reduced malaria vector density in metal-roof houses compared to thatch-roof houses has been reported in several studies, including a Tanzanian study where metal-roof houses had 33% less *Anopheles arabiensis* than thatch-roof houses [30], and a Ugandan study where there were 38–43% fewer *An. gambiae s.l*. in metal-roof houses [31]. Results are, however, contradictory in other studies. In The Gambia, metal-roof houses were not associated with fewer mosquitoes [6], and in an experimental study, metal roof houses with closed eaves and mud walls had similar numbers of mosquitoes as thatch-roofed houses with open eaves and mud walls [7]. It may be that there is a trade off between the killing effect of metal roofs due to the hostile indoor climate, and a heating effect of the roof, which can increase carbon dioxide production from humans and therefore attract more malaria vectors [7, 32].

Ultimately, whether a metal-roof house has more or less mosquitoes than a thatch-roof house will depend on how porous the house is to mosquitoes and the extent of ventilation [16]. Further research is needed to understand the importance of different house construction features on indoor climate and vector entry.

The study has several limitations. Firstly, ITN use the previous night was assessed by asking the caregiver, which may be prone to social desirability bias [33]. The use of an ITN will usually vary over the transmission season, but we only measured use during the baseline survey. This may have impacted on our ability to identify an association between ITN usage and indoor density of malaria vectors. Secondly, the study did not collect information on all possible risk factors for malaria vector house entry. For example, whether the doors were kept open until late in the evening was not included in the risk factors assessed. The analysis did not adjust for unmeasured risk factors, which may have confounded the associations observed.

The cohort study in which this entomological study was nested did not identify strong risk factors for *P. falciparum* infection, with only overnight travel and higher SES factor score being associated with higher rates of *P. falciparum* infection [22]. It is difficult to reconcile the entomological and epidemiological findings and further studies are needed. It is perhaps unsurprising that the risk factors for malaria vector density and *P. falciparum* infection in children differed, since higher indoor vector density does not automatically imply higher infection risk. The indoor density of malaria vectors may be less important in this study area due to the observation of increasing outdoor biting with some studies suggesting ~ 54% of *An. gambiae s.l.* host seeking outdoors [34] or more simply, it does not accurately reflect the transmission intensity experienced by a child sleeping under a net. Research also suggests that the study communities spend more time outside in the peri-domestic environment during peak biting times than previously thought [35].

What are the implications of the study findings for vector control and future research? The study highlights the potential of improved housing to reduce malaria transmission and supports the results of systematic reviews and multi-country research studies on this topic [36, 37]. Housing improvements tend to be implemented as a package and, in line with this, our study found that metal-roof sleeping spaces were more likely to have floors and walls made of finished materials than thatch-roof sleeping spaces. Improving house construction should be a focus for malaria reduction, with increasing evidence in support of screened, self-closing doors, closed eaves, raising buildings off the ground, screened windows on either side of building for ventilation and solid roofs [16, 38, 39]. As well as contributing to the development agenda, there is also evidence that improved housing can reduce risk of other major causes of death in children including diarrhoea, growth failure and anaemia [40]. While other vector control tools such as dual-active ingredient ITNs are now being deployed in the study area, the study results highlight the importance of non-insecticidal interventions such as house improvement to increase long-term resilience against malaria and for insecticide resistance management.

## Conclusion

This study in south-west Burkina Faso demonstrates a 45% reduction in indoor density of malaria vectors in sleeping spaces with a metal roof compared to those sleeping spaces with thatch roofs. The study adds to the growing evidence base supporting the use of housing improvement against malaria.

## Supplementary Information

**Additional file 1:** Questionnaire administered to head of household on asset ownership and household characeristics.

## Data Availability

The datasets used and/or analysed during the current study are available from the corresponding author on reasonable request.

## Abbreviations

CI: Confidence interval
CSP: Circumsporozoite protein
DHS: Demographic and Health Survey
ITN: Insecticide treated-net
IRR: Incidence rate ratio
PCR: Polymerase chain reaction
SES: Socio-economic status
