# Supplementary material for: Risk factors associated with house entry of malaria vectors in an area of Burkina Faso with high, persistent malaria transmission and high insecticide resistance
Source: Malar J. 2021 Oct 10;20:397. doi: 10.1186/s12936-021-03926-5 (PMC8504047; doi:10.1186/s12936-021-03926-5)
Supplement: Supplementary file 1 — Additional file 1: Questionnaire administered to head of household on asset ownership and household characeristics. [file 12936_2021_3926_MOESM1_ESM.docx]

**Additional Information: File 1: Questionnaire administered to head of household on asset ownership and household characeristics**

**MALARIA INDICATOR SURVEY**

**HOUSEHOLD QUESTIONNAIRE ON SOCIO-ECONOMIC STATUS**

BURKINA FASO

CENTRE NATIONAL DE RECHERCHE ET DE FORMATION SUR LE PALUDISME (CNRFP)

| **A: IDENTIFICATION** | |
| --- | --- |
| NAME OF PLACE ___________________________________  NAME OF HOUSEHOLD HEAD _______________________________  CLUSTER NUMBER . . . . . . . . . . . . . . . . . . . . . . . . . . . . . .  CONCESSION NUMBER. . . . . . . . . . . . . . . . . . . . . . . . . .  HOUSEHOLD NUMBER . . . . . . . . . . . . . . . . . . . . . . . . . . . .  ADMINISTRATIVE REGION……………………………………………  SETTING (URBAIN = 1, RURAL = 2) . . . . . . . . . . . . . . . . . . | \|  \|  \|  \| \| --- \| --- \| --- \|   CLUSTER   \|  \|  \| \| --- \| --- \|   CONCESSION   \|  \|  \| \| --- \| --- \|   HOUSEHOLD     \|  \|  \| \| --- \| --- \|   REGION     \|  \|  \| \| --- \| --- \|   SETTING |

| **B: INVESTIGATOR VISITS** | | | | | | |
| --- | --- | --- | --- | --- | --- | --- |
|  | 1 | | 2 | 3 | | VISITE FINALE |
| DATE  INTERVIEWER’S NAME  RESULT |  | |  |  | | \|  \|  \| \| --- \| --- \|   DAY   \|  \|  \| \| --- \| --- \|     MONTH   \|  \|  \|  \|  \| \| --- \| --- \| --- \| --- \|   YEAR   \|  \|  \|  \| \| --- \| --- \| --- \|   INT. NO   \|  \|  \| \| --- \| --- \|   RESULT |
| NEXT VISIT DATE:    TIME: |  | |  |  | | TOTAL NUMBER OF VISITS |
| *RESULT CODES:  1 COMPLETED  2 NO HOUSEHOLD MEMBER AT HOME OR NO COMPETENT MEMBER AT HOME AT TIME OF VISIT  3 ENTIRE HOUSEHOLD ABSENT FOR EXTENDED PERIOD 4POSTPONED  5 REFUSED  6 DWELLING VACANT OR ADDRESS NOT A DWELLING 7 DWELLING DESTROYED  8 DWELLING NOT FOUND  9 OTHERS  ____________________________________________________  (PRÉCISER) | | | | | | TOTAL PERSONS IN HOUSEHOLD  TOTAL  ELIGIBLE WOMEN  LINE NUMBER OF  RESPONDENT TO HOUSEHOLD QUESTIONNAIRE |
| SUPERVISOR  NAME ______________ | | FIELD EDITOR  NAME ________________ | | | KEYED BY  NAME ______________ | |

**C: INTRODUCTION AND CONSENT**

Hello my Name Is……………………………………………………………………………. I work for the CNRFP, a government agency under the Ministry of Health. The CNRFP conducts research to learn more about health and disease in Burkina Faso. The CNRFP and its partners are trying to learn more about people's health experiences. We are NOT physicians but are interested in knowing how to help strengthen health services for all profits in the future.

To do this, we would like to collect information on the possessions of the households to categorize them according to their level of possession of these goods. The purpose of this collection is the dynamics of the search for obstacles that may impede access to prevention and treatment of malaria.

Households involved in the sample for the survey may decide to discontinue their participation during the collection. They are free to withdraw at any time, without penalty and / or consequences.

SIGNATURE OF

INTERVIEWER ………………………………..DATE:……………………………………………………..

RESPONDENT AGREES TO BE INTERVIEWED 1

RESPONDENT REFUSES TO BE INTERVIEWED 2 END

**D: SES SURVEY MODULE QUESTIONS**

| LINE NO (MIS 2014) | QUESTIONS AND FILTERS | CODES | SKIP |
| --- | --- | --- | --- |
|  | How many people slept in this household last night? | \|  \|  \| \| --- \| --- \|   Enter number. If unknown enter 98 |  |
| 102 | What is the main source of drinking water for members of your household? | **PIPED WATER**  PIPED INTO DWELLING 11  PIPED INTO YARD/PLOT 12  PIPED INTO NEIGHBOUR 13  PUBLIC TAP/STANDPIPE 14  TUBE WELL OR BOREHOLE 21  **DUG WELL**  PROTECTED WELL 31  UNPROTECTED WELL 32  **WATER FROM SPRING**  PROTECTED SPRING 41  UNPROTECTED SPRING 42  RAINWATER 51  TANKER TRUCK 61  CART WITH SMALL TANK 71  SURFACE WATER (RIVERS/DAMS/ 81  LAKES/POND/STREAM/IRRIGATION CANAL  BOTTLED WATER 91  OTHER _______________________________96  (SPECIFY) | 107  107 |
| 103 | Where is the water source located? | IN OWN DWELLING 1  IN OWN YARD/PLOT 2  ELSEWHERE 3 | 107 |
| 107 | What kind of toilet facility do members of your household usually use? | **FLUSH OR POUR FLUSH TOILET**  FLUSH TO PIPED SEWER SYSTEM 11  FLUSH TO SEPTIC TANK 12  FLUSH TO PIT LATRINE 13  FLUSH TO SOMEWHERE ELSE 14  FLUSH, DON’T KNOW WHERE 15  **PIT LATRINE**  VENTILATED IMPROVED PIT LATRINE 21  PIT LATRINE WITH SLAB 22  PIT LATRINE WITHOUT SLAB/OPEN PIT 23  COMPOSTING TOILET 31  BUCKET TOILET 41  HANGING TOILET/LATRINE 51  NO FACILITY/BUSH/FIELD 61  OTHER _______________________________96  (SPECIFY) | 110 |
| 108 | Do you share this toilet facility with other households? | YES 1  NO 2 | 109  110 |
| 109 | Including your own household, how many households use this toilet facility? | NOMBRE DE MÉNAGES  0  SI MOINS DE 10  10 MÉNAGES OU PLUS 95  NE SAIT PAS 98 |  |
| 110 | Does your household have?  Electricity?  A Radio?  A Television?  A mobile phone?  A non-mobile phone?  A refigerator?  A table?  A chair?  Cupboard/Library?  Cooker/Microwave?  Freezer?  Hunter Gun?  Plow? | OUI NON  ELECTRICITY 1 2  RADIO 1 2  TELEVISION 1 2  MOBILE TELEPHONE 1 2  NON-MOBILE TELEPHONE 1 2  REFRIGERATOR 1 2  TABLE 1 2  CHAIR 1 2  CUPBOARD/LIBRARY 1 2  COOKER/MICROWAVE 1 2  FREEZER 1 2  HUNTER GUN 1 2  PLOW 1 2 |  |
| 111 | What type of fuel does your household mainly use for cooking? | ELECTRICITY 01  LPG 02  CHARCOAL 07  WOOD 08  NO FOOD COOKED IN HOUSEHOLD 95  OTHER_______________________________96  (SPECIFY) |  |
| 114 | OBSERVE MAIN MATERIAL OF THE FLOOR OF THE DWELLING.  RECORD OBSERVATION. | **NATURAL FLOOR**  EARTH/SAND 11  DUNG 12  **FINISHED FLOOR**  CERAMIC TILES 33  CEMENT 34  OTHER ___________________________­­­­­­___96  (SPECIFY) |  |
| 115 | "OBSERVE MAIN MATERIAL OF THE ROOF OF THE DWELLING.  RECORD OBSERVATION." | **NATURAL ROOFING**  THATCH/PALM LEAVES 12  **FINISHED ROOFING**  METAL 31  OTHER______________________________96  (SPECIFY) |  |
| 116 | "OBSERVE MAIN MATERIAL OF THE EXTERIOR WALLS OF THE DWELLING.  RECORD OBSERVATION." | **NATURAL WALLS**  CANE/PALM/TRUNKS 12  DIRT 13  **RUDIMENTARY WALLS**  BAMBOO/WOOD WITH MUD 21  STONES WITH MUD 22  UNCOVERED ABODE 23  **FINISHED WALLS**  CEMENT 31  STONES WOTH LIME/CEMENT 32  BRICKS 33  CEMENT BLOCKS 34  COVERED ABODE 35  WOOD PLANKS/SHINGLES 36  OTHER_______________________________96  (SPECIFY) |  |
| 117 | How many rooms in this household are used for sleeping? | \|  \|  \| \| --- \| --- \|   ROOMS  UNDER TREE / FREE AIR 95 |  |
| 118 | Does any member of this household own:?  A Canoe?  A watch?  A bicycle?  A motorcycle/ scooter?  An animal drawn cart?  A car or truck? | OUI NON  CANOE 1 2  WATCH 1 2  BICYCLE 1 2  MOTORCYCLE/SCOOTER 1 2  ANIMAL DRAWN CART 1 2  CAR/TRUCK 1 2 |  |
| 119 | Does any member of your household own any agricultural land? | YES 1  NO 2 | 120  121 |
| 120 | How many hectares of agricultural land do members of this household own?  IF 95 OR MORE, CIRCLE '950'. | HECTARES (ONE DECIMAL POINT)   \|  \|  \|  \| \| --- \| --- \| --- \|     95 HECTARES OR MORE 950  DON’T KNOW 998 |  |
| 121 | Does this household own any livestock, herds, other farm animals, or poultry? | YES 1  NO 2 | 122  123 |
| 122 | How many of the following animals does this household own?    "IF NONE, RECORD '00'.  IF 95 OR MORE, RECORD '95'.  IF UNKNOWN, RECORD '98'." | \| COWS/BULLS \|  \|  \| \| --- \| --- \| --- \| \| HORSES/DONKEY/MULES \|  \|  \| \| GOATS \|  \|  \| \| PIGS \|  \|  \| \| SHEEP \|  \|  \| \| CHICKEN/DUCKS \|  \|  \| |  |
| 123 | Does any member of this household have a bank account? | YES 1  NO 2 |  |
